# Prostate cancer treated by anti-androgens: is sexual function preserved?

**DOI:** 10.1054/bjoc.1999.0916

**Published:** 2000-01-18

**Authors:** F H Schröder, L Collette, T M de Reijke, P Whelan, members of the E. O. R. T. C. Genitourinary Group

**Affiliations:** Department of Urology, Erasmus University and Academic Hospital and the European Organization for Research and Treatment of Cancer (EORTC) Genitourinary Group, PO Box 1738, Rotterdam, DR, 3000, The Netherlands

**Keywords:** prostate cancer, anti-androgens, sexual function

## Abstract

This paper reports on results of the EORTC protocol 30892, an open, prospective, randomized study of 310 patients with previously untreated metastatic prostate cancer with favourable prognostic factors who were treated by either flutamide (FLU) or cyproterone acetate (CPA) monotherapy. The final analysis with regard to the main end points, time to progression and survival are still pending. Final results related to the evaluation of sexual functioning prior to and under treatment are reported here. Of 310 randomized patients 294 were eligible for evaluation within this side study. The median age was 71 years (range 48–85). Potential risk factors related to age, general health and prostate cancer were evaluated. For evaluation of sexual functions a five-item questionnaire was used which was administered by the investigator. The protocol allowed time dependent observations at 3-monthly follow-up visits. Sexual functioning was dependent on age but not on prostate cancer-related parameters. Sexual functions at entry were similar within the two treatment groups, spontaneous (nightly) erections and sexual activity were seen in 43–51% and 29–35% of cases. Under treatment, sexual functions under FLU and CPA declined slowly with median times of 12.9 and 5.8 months versus 13.7 and 8.9 months respectively for spontaneous erections and sexual activity. Eventually, with an average observation time in excess of 2 years, loss of spontaneous erections and of sexual activity occurred in 80% versus 92% and in 78% versus 88% of men under FLU versus CPA treatment respectively. None of these differences reached statistical significance. Maintenance of potency under treatment with FLU as reported in the literature is not confirmed in this study. However, loss of sexual functions under monotherapy with both antiandrogens is slow and 10–20% of men retain sexual activity after 2–6 years of treatment. This observation can be exploited in new treatment schemes and is likely to lead to improved quality of life. The advantage of FLU in time and total preservation of sexual functions is statistically not significant and must be balanced against the side effects of FLU and other pure antiandrogens, which may exceed those of CPA especially with respect to gynaecomastia. Hepatic toxicity may limit the long-term use of both drugs. © 2000 Cancer Research Campaign


*British Journal of Cancer* (2000) **82**(2), 283–290
© 2000 Cancer Research Campaign
Article no. bjoc.1999.0916

### Prostate cancer treated by anti-androgens: is sexual function preserved?

**FH Schröder, L Collette, TM de Reijke, P Whelan and members of the EORTC Genitourinary Group***

Department of Urology, Erasmus University and Academic Hospital, PO Box 1738, 3000 DR Rotterdam, The Netherlands, and the European Organization for Research and Treatment of Cancer (EORTC) Genitourinary Group

**Summary** This paper reports on results of the EORTC protocol 30892, an open, prospective, randomized study of 310 patients with previously untreated metastatic prostate cancer with favourable prognostic factors who were treated by either flutamide (FLU) or cyproterone acetate (CPA) monotherapy. The final analysis with regard to the main end points, time to progression and survival are still pending. Final results related to the evaluation of sexual functioning prior to and under treatment are reported here. Of 310 randomized patients 294 were eligible for evaluation within this side study. The median age was 71 years (range 48–85). Potential risk factors related to age, general health and prostate cancer were evaluated. For evaluation of sexual functions a five-item questionnaire was used which was administered by the investigator. The protocol allowed time dependent observations at 3-monthly follow-up visits. Sexual functioning was dependent on age but not on prostate cancer-related parameters. Sexual functions at entry were similar within the two treatment groups, spontaneous (nightly) erections and sexual activity were seen in 43–51% and 29–35% of cases. Under treatment, sexual functions under FLU and CPA declined slowly with median times of 12.9 and 5.8 months versus 13.7 and 8.9 months respectively for spontaneous erections and sexual activity. Eventually, with an average observation time in excess of 2 years, loss of spontaneous erections and of sexual activity occurred in 80% versus 92% and in 78% versus 88% of men under FLU versus CPA treatment respectively. None of these differences reached statistical significance. Maintenance of potency under treatment with FLU as reported in the literature is not confirmed in this study. However, loss of sexual functions under monotherapy with both antiandrogens is slow and 10–20% of men retain sexual activity after 2–6 years of treatment. This observation can be exploited in new treatment schemes and is likely to lead to improved quality of life. The advantage of FLU in time and total preservation of sexual functions is statistically not significant and must be balanced against the side effects of FLU and other pure antiandrogens, which may exceed those of CPA especially with respect to gynaecomastia. Hepatic toxicity may limit the long-term use of both drugs. © 2000 Cancer Research Campaign

**Keywords**: prostate cancer; anti-androgens; sexual function

The data presented in this report describe the results of the first evaluation of sexual potency obtained within a prospective, open, randomized study of previously untreated patients with metastatic prostate cancer (EORTC protocol 30892). Cyproterone acetate (CPA) as standard treatment and with a well established castration-like effect on libido and potency (Ahrens, 1990) is compared with flutamide (FLU), which is considered to preserve libido and potency (Sogani et al, 1984; Lund and Rasmussen, 1988; Boccon-Gibod, 1998). The European Organization for Research and Treatment of Cancer (EORTC) study 30892 was set up to compare the effectiveness of monotherapy with flutamide versus CPA in men with metastatic prostate cancer with favourable prognostic factors. An additional goal was to explore the possibility of utilizing less aggressive (minimally invasive?) endocrine treatment of prostate cancer patients, which would improve quality of life under treatment for those who are potent and sexually active and wish to remain so. The main end points of this study, which have not yet been reached and which are not subject to this report, are time to progression, cancer-specific and overall survival. The ‘soft approach’ of anti-androgen monotherapy was chosen on the background of negative findings of the EORTC Genitourinary (GU) Group with relation to the use of maximal androgen blockade (MAB) (Robinson et al, 1995; Voogt et al, 1998). Marginal, but no significant benefit of MAB has recently been shown by a metaanalysis and no benefit was seen in a large American study (Dalesio et al, 1995; Eisenberger et al, 1998).

*Received 5 March 1999*
*Revised 29 July 1999*
*Accepted 5 August 1999*

*Correspondence to:* FH Schröder

### MATERIALS AND METHODS

### Evaluation of potency

Evaluation of potency and sexual activity was carried out at entry, at 3-monthly visits during the first 2 years of treatment and 6-monthly thereafter until progression, by means of a physician-administered questionnaire. Five questions were asked. The questions were supplied in English to all participating centres, which were located in the UK, The Netherlands, Italy, Belgium, Turkey and six other European countries. The questions had to be translated ad hoc by the treating physician and asked in the patients’ mother tongue. The English phrasing of the questions was as follows:

*Major contributors: M Pavone-Macaluso, Palermo; J Mattelaer, Kortrijk; RPF van Velthoven, Brussels; DWW Newling, Amsterdam; UE Studer, Bern; M Brausi, Carpi; A Akdas, Istanbul; L Denis, Antwerp; M de Pauw, Brussels

1. Did you notice an erect penis sometimes during the night or when waking up in the last 3 months?
2. Do you consider yourself sexually active in some way?
3. Do you have an erection with sexual excitement?
4. Do you reach an orgasm during sexual activity?
5. Do you ejaculate with sexual activity?

Questions 3, 4 and 5 were only to be asked if question 2 was answered with ‘yes’. Throughout this paper the term ‘potent’ is applied for men who answered ‘yes’ to questions 2 and/or 1.

### The questions

This protocol was designed during 1988. The questions were selected and modified from questionnaires proposed by Frenken (1978). These questionnaires were the most advanced source validated instruments available at that time. The translations and the combination of the simplified list of five questions used in this protocol were not validated.

In selecting the questions it was considered that older men often do not have a partner. They may be sexually active in some way without actually having intercourse. It seemed to be in the interest of the accuracy of the evaluation to include such situations. For this reason, the obvious question: ‘Are you able to have sexual intercourse?’ was not included. The basic thought was that organic functioning and libido could best be assessed by questions 1 and 2. This included the possibility that a man might be sexually active without actually having erections. For this reason, for those men who answered question 2 with yes, further specification is requested in questions 3, 4 and 5.

### Definitions

The following eligibility rules for evaluation were established:

- Patients who answered ‘yes’ to question 1 and/or 2 (for potency at entry) and who had at least one follow-up form available were included in the evaluation of potency at entry and in the time-dependent analysis.
- *Recovery of potency*: Some patients answered ‘no’ on question 1 and/or 2 at entry, but ‘recovered’ potency later on. They were considered to be potent at entry and were included in the time-dependent analysis.
- *Time to definite disability*: Time to definite disability of a given function is defined as the time from entry on study to the date of definitive loss of the function, which is defined as the date of the first reported ‘no’, which is not followed by a ‘yes’ to the given question at any subsequent follow-up visit. The time to definitive disability of each function has been analysed as a time to event function using Kaplan–Meier curves.
- *Transient disability of the function* is defined as any ‘no’ reported by the patient, irrespective of his answers to the question at subsequent follow-up visits. Thus, the expression ‘transient loss’ includes those men who lost a given function definitively or temporarily. The frequency of definitive loss of functions and the frequency of transient loss with inclusion of transient loss are separately calculated for the two treatment arms.

### Statistics

The original sample size calculation of protocol 30892 was based on time to progression, cancer specific survival and overall survival, a sample size calculation related to the potency outcome was not done.

Percentages were compared by the use of the $\chi^2$ test (*) (Agresti, 1990) or the $\chi^2$ test for linear trend (**) (Armitage, 1995) whenever the variables had more than two ordered categories. Standard Kaplan–Meier curves were used to estimate the time to the loss of sexual functions (Kaplan and Meier, 1958). These were compared using the log-rank test (***) (Mantel, 1966). All tests were two-sided and the 0.05 significance level was used.

### RESULTS

The overall rate of sexual potency was rather low. A total of 138 of 294 men (47%) claimed to have morning erections at entry and only 94/294 (32%) were sexually active.

### Eligibility

Of the 310 patients, 294 were eligible for the evaluation of potency at entry into the study. Two-hundred and seventy-eight men with follow-up information regarding potency are eligible to the evaluation of loss of sexual functions and of their time-dependent evaluation. The lack of follow-up of a total of 33 men was due to incomplete patient cooperation as well as other factors.

#### *Baseline characteristics*

Baseline characteristics of patients were evaluated with respect to morning/night erections (question 1) and sexual activity (question 2) at entry. These characteristics included: age, performance status, chronic associated disease, previous prostate surgery and other treatment, the TNM categories and the grade of differentiation. An attempt was made to identify characteristics as possible risk factors for the loss of sexual functions. As one would expect, the proportion of men who have nightly or morning erections and who are sexually active decreases with age ($P$ = 0.001). A decreased WHO performance status seems to have a similar impact though the effect does not reach statistical significance. All characteristics did not have any impact (original data not shown).

The baseline patient characteristics were also compared between the treatment arms. It appeared that the patients entered on the CPA arm were significantly younger than those on the FLU arm ($P$ = 0.003) with a median age of 69 years (range 51–85) as compared to 73 years (range 48–85) on the FLU arm. This needs to be taken into account when comparing potency between the two treatment arms since age is a strong predicting factor for potency.

At the time of this evaluation only 62 of the 278 eligible patients were still on study and under treatment. Of these, 28 were sexually active at base-line and those contribute to the analysis of potency. Eight of these 28 were still sexually active in some way at the time of their last follow-up visit. The only additional information that could be obtained would be an update on these eight patients, who might or might not lose their potency. One may expect that this will not change any of the figures used in the comparisons. Further follow-up on these eight men would not change the final conclusions of the paper. For these reasons this evaluation is considered definite.

 © 2000 Cancer Research Campaign

**Table 1** Potency at entry or recovery under treatment (if not functioning at entry)

| | | FLU *n* = 147 *N* | (%) | CPA *n* = 147 *N* | (%) | *P*-value |
|---|---|---|---|---|---|---|
| 1. | Morning erections | 54 | (36.7) | 61 | (41.5) | 0.409 |
| | Recovery | 9 | (6.1) | 14 | (9.5) | 0.386 |
| | Total[a] | 63 | (42.9) | 75 | (51.0) | 0.161 |
| 2. | Sexually active | 38 | (25.9) | 40 | (27.2) | 0.579 |
| | Recovery | 5 | (3.4) | 11 | (7.5) | 0.123 |
| | Total[a] | 43 | (29.3) | 51 | (34.7) | 0.317 |
| 3. | Erections with sexual excitement | 36 | (24.5) | 37 | (25.2) | 0.893 |
| | Recovery | 6 | (4.1) | 10 | (6.8) | 0.304 |
| | Total[a] | 42 | (28.6) | 47 | (32.0) | 0.794 |
| 4. | Orgasm present | 37 | (25.2) | 38 | (25.9) | 0.894 |
| | Recovery | 4 | (2.7) | 8 | (5.4) | 0.238 |
| | Total[a] | 41 | (27.9) | 46 | (31.3) | 0.523 |
| 5. | Ejaculation present | 34 | (23.1) | 34 | (23.1) | 0.999 |
| | Recovery | 4 | (2.7) | 5 | (3.4) | 0.735 |
| | Total[a] | 38 | (25.9) | 39 | (26.5) | 0.447 |

[a]These patients are included in the time course evaluation per treatment but only if at least one follow-up form was available.

**Table 2** Definite and transient loss of potency in men functioning at entry or with recovery of function under treatment per treatment regimen

| | | FLU *n* = 136 *n* (%) | CPA *n* = 142 *n* (%) | *P*-value |
|---|---|---|---|---|
| 1. Morning erections | Definitive loss | 43/60 (71.7) | 5910/75 (78.7) | 0.347 |
| | Transient loss | 48/60 (80.0) | 69/75 (92.0) | 0.042 |
| 2. Sexually active | Definitive loss | 31/41 (75.6) | 36/51 (70.6) | 0.590 |
| | Transient loss | 32/41 (78.1) | 45/51 (88.2) | 0.189 |
| 3. Erections with sexual excitement | Definitive loss | 35/40 (87.5) | 36/47 (76.6) | 0.191 |
| | Transient loss | 36/40 (90.0) | 41/47 (87.2) | 0.687 |
| 4. Orgasms | Definitive loss | 33/39 (84.6) | 36/46 (78.3) | 0.455 |
| | Transient loss | 34/39 (87.2) | 44/46 (95.6) | 0.157 |
| 5. Ejaculation | Definitive loss | 32/36 (88.9) | 32/39 (82.1) | 0.586 |
| | Transient loss | 32/36 (88.9) | 38/39 (97.4) | 0.138 |

Questions 3–5 only to be asked if the answer to question 1 was ‘yes’. Only men with at least one follow-up form are included.

### Potency at entry

Potency at entry is assumed if questions 1 and/or 2 were answered positively initially or if positive answers were obtained during the course of treatment after initial denial. Table 1 indicates potency at entry or recovery of functions under treatment by FLU or CPA according to our definitions. It can be seen that the distribution of sexual functioning in the different categories between the two treatment arms is rather similar at entry, no statistically significant differences are encountered. As already mentioned questions 3–5 were asked only if question 2 was answered positively. The results can therefore be read as follows: in, for example the FLU group, 43 patients were considered to be sexually active, of these 42 experienced erections with sexual excitement, 41 were able to reach an orgasm and 38 ejaculated. The small differences seen in totals between the treatment groups are not statistically significant. More patients recovered under treatment with CPA than with FLU. Again, the numbers do not reach statistical significance. Some of these patients recovered late after their entry on study, though for the majority the recovery occurred within 1 year of entry on study. Including these patients in the time-dependent evaluation of potency may slightly bias the results towards a decrease of the difference between FLU and CPA. Also, the fact that the patients were slightly younger on CPA is likely to bias the comparison in the same way. The analysis was repeated including only those who were ‘potent’ at entry (and with exclusion of those who recovered ‘potency’), and also with a stratification for age. The conclusions did not change, no statistical significance was encountered.

### *Time-dependent evaluation*

Table 2 indicates the proportion of those who lost one of five sexual functions under study during the follow-up under treatment. Definite and transient loss of functions are defined in the methods sections.

The totals of those of whom sexual functions are reported in Table 1 are taken over as reference values in Table 2 after excluding those men who did not have at least one follow-up form available. This correction explains the small differences in the total number of patients considered for each question as compared to Table 1. This situation occurred only in the FLU arm.

**Table 3** Time to definitive loss of sexual functions per treatment arm (all patients with activity at baseline or recovery)

| | | Observed diagnosis/ total numbers | Median (months) | (95% CI) | *P*-value |
|---|---|---|---|---|---|
| 1. | Morning erections | | | | |
| | FLU | 43/60 | 12.9 | 9.9–23.4 | |
| | CPA | 59/75 | 5.8 | 3.5–12.6 | = 0.154 |
| 2. | Sexually active | | | | |
| | FLU | 31/41 | 13.7 | 8.8–21.4 | |
| | CPA | 36/51 | 8.9 | 3.6–17.0 | = 0.907 |
| 3. | Erections with sexual excitement | | | | |
| | FLU | 35/40 | 10.0 | 3.6–15.8 | |
| | CPA | 36/47 | 5.8 | 2.4–16.5 | = 0.684 |
| 4. | Orgasm | | | | |
| | FLU | 33/39 | 8.8 | 3.6–15.8 | |
| | CPA | 36/46 | 8.3 | 2.5–16.5 | = 0.616 |
| 5. | Ejaculation | | | | |
| | FLU | 32/36 | 4.0 | 2.7–15.8 | |
| | CPA | 33/39 | 3.1 | 1.8–8.9 | = 0.924 |

The set up of the study allows to evaluate sexual functions in a time-dependent fashion. At the time of this evaluation the median follow-up amounts to 3.3 years for potency and to 4.5 years for the survival status. The difference in duration of follow-up relates to the evaluation of the potency status being stopped whenever the patients stop the protocol treatment.

The following observations are made: in the FLU group fewer patients lost morning erections and sexual activity than in the CPA group. The difference with respect to morning erections is statistically significant ($P = 0.042$). Significance is not reached for the difference in loss of sexual activity (78.1 vs 88.2%). Small differences with regard to questions 3, 4 and 5 again turn out not to be statistically significant. It is remarkable that with prolonged treatment morning erections and sexual activity are preserved under FLU in 20.0 and 21.9% and also with CPA in 8 and 11.8% respectively. The same analysis on only the patients who are positive at entry, excluding those who recovered under treatment gave similar results.

In Table 3 the time to the definitive loss of sexual functions is summarized per treatment group. Again, there are no significant differences. However, there seems to be a trend towards a longer preservation of morning erections and sexual activity in men who use FLU. The median times to loss of morning erections and sexual activity amount to 12.9 months and 13.7 months under FLU and to 5.8 and 8.9 months under CPA. These differences, however, are not significant, probably because of small numbers and wide confidence intervals. Median times in this context indicate the time until 50% of all men have lost the respective function. Loss of ejaculation is reported much earlier than the loss of all other functions under study.

When the analyses were repeated with exclusion of patients who answered negatively at baseline and recovered under treatment the same conclusions were reached. Because the observed difference in the distribution of the age of the patients between the two groups may introduce a bias in the comparisons, analyses stratified for age were also carried out, again leading to similar conclusions.

A consort diagram (Figure 1) indicates the flow of events related to question 2 (sexually activity) per treatment arm.

Figure 2A–D further illustrates these findings. The most important observation may be that loss of sexual functions rarely occurs abruptly, but in most patients within a year from start of treatment. There is a trend toward a more favourable time course under FLU treatment than under CPA. Also, it is interesting that some patients retain their sexual functions for long time periods lasting between 2 and 6 years. Most projections level off between 10 and 20%. None of the Kaplan–Meier projections, which indicate the time course of change for each question reveal a statistically significant advantage of one of the treatment group above the other. Adjusting the analysis for the influence of progressive disease did not change the conclusions.

### DISCUSSION

At the time this study was set up (1988), a literature review did not reveal any measuring instrument for sexual potency that was specifically designed for the intended study. The questionnaire from which most of the questions were adapted (Frenken, 1978)

**Randomization ($n = 310$)**

**Eligible (294)**

| ***Questions*** | ***FLU (147)*** ***n* (%)** | ***CPA (147)*** ***n* (%)** |
|---|---|---|
| Sexually active at entry | 38 (25.8) | 40 (27.2) |
| Recovery | 5 (3.4) | 11 (7.5) |
| Total active | 41 (27.9) | 51 (34.7) |
| Transient loss (only applicable if potent at entry) | 32 (78.1)* | 45 (88.2)* |
| Definite loss under treatment | 31 (75.6)* | 36 (70.6)* |
| Median time to loss (months) | 13.7 | 8.9 |

* % of all active at entry.

**Figure 1** Consort diagram relating to sexual activity (Question 2) at entry and during/after treatment by cyproterone acetate or flutamide

© 2000 Cancer Research Campaign

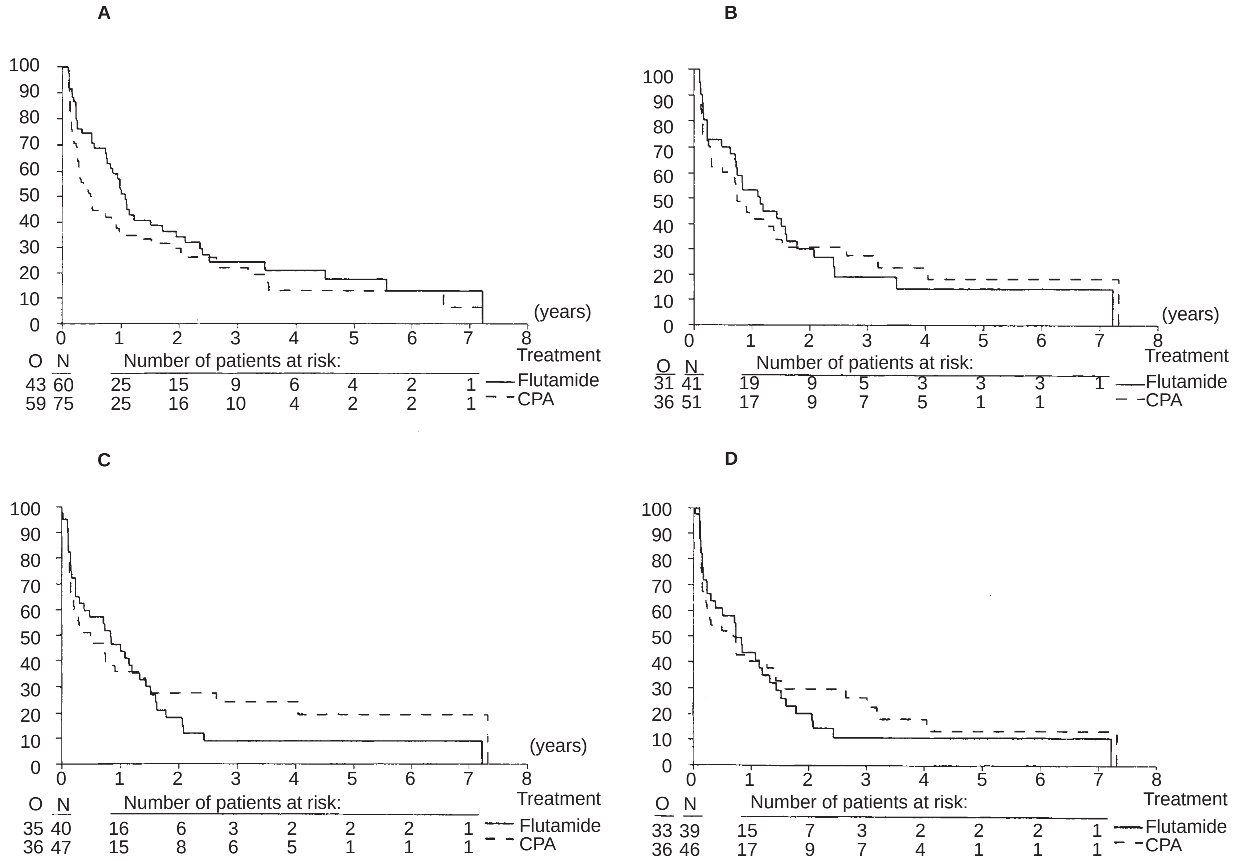

**Figure 2** Kaplan–Meier projections of time to loss of sexual functions (Questions 1–4). Patients recovering functions during treatment are indicated. (**A**) morning/night erections, *P* = 0.154; (**B**) sexual activity, *P* = 0.907; (**C**) erections with sexual excitement, *P* = 0.684; (**D**) orgasm, *P* = 0.616

was at that time validated within The Netherlands and for population-based but not disease-related studies. At the time of the design of this protocol the EORTC-GU group had to settle for a strongly simplified and still effective set of questions, which could be adapted to a multicentre setting, the involvement of multiple nationalities, the need for providing one protocol in the English language and the logistic difficulty of providing validated translations of the questionnaire in the 11 languages of the countries involved in this protocol, which would have been necessary if a patient administered questionnaire had been considered. The purpose was to design questions that would measure organic sexual function (question 1), libido combined with sexual capabilities (question 2) and sexual satisfaction (questions 3, 4 and 5) of those who were sexually active. Only in recent years more elaborate measuring instruments have been developed (Feldman et al, 1994; Fitzpatrick et al, 1998) such as the nine-item questionnaire used in the Massachusetts Male Aging Study (MMAS), and within a Swedish study comparing general age matched population to prostate cancer cases untreated and under various forms of treatment (Helgason et al, 1998). Obviously, these efforts to develop measuring instruments have all led to different questionnaires and different methodology of evaluation so that literature-based comparisons are very difficult.

### Potency at entry

Within this report, males who claim to have spontaneous erections at night or in the morning and/or those who claim to be sexually active in some way, are considered to be potent. The presence of spontaneous erections is often but not always associated with the desire of being sexually active. Potency at entry into the study (spontaneous erections or sexual activity) was reported in 138/294 (46.9%). Ninety-four out of 294 (32.0%) men included those who scored negative for these questions initially but regained sexual functions during the course of treatment. The study does not provide a control group that would allow judgement on the impact of prostate cancer with respect to sexual functioning of men of a similar age without prostate cancer. In agreement with the MMAS (Feldman et al, 1994) and the data provided by Helgason et al (1998) this study showed that potency is strongly age-related. The MMAS study differentiates between minimal, moderate and complete loss of potency. A total of 1290 of 1707 men aged 40–70 provided answers to a self administered nine-item questionnaire. A comparison with the data obtained in the present study is impossible. However, even considering a very much younger average age, the combined prevalence of minimal, moderate and complete impotence was 52%. Among men aged 65–70 years the combined

*© 2000 Cancer Research Campaign* 

rate of moderate and complete impotence was about 53%. The Swedish study (Helgason et al, 1998) reports on ‘any decrease of sexual function’ with time in 342 men with untreated prostate cancer compared to an age-matched cohort of 314 men without prostate cancer. Loss of sexual desire, erectile function and orgasm was reported by 51, 77 and 71% in men without prostate cancer and by 75, 90 and 83% in men with prostate cancer. Morley (1988) found a rate of impotence of 27% in men more than 50 years old undergoing a general health screening. Diokno et al (1990) reported 40% impotence in 283 men who were older than 60 years.

Many of the participants were aware of the diagnosis of prostate cancer prior to entry into this protocol for various periods of time. This may explain why some men had lost all sexual interest and felt that they might have become impotent at entry. With the usual remission of the disease under endocrine treatment and due to other accompanying circumstances such as a new partner, sexual interest and potency have returned in some as indicated in Tables 1 and 2.

### Time-dependent observations

Loss of potency under treatment with anti-androgens, pure (FLU) or steroidal (CPA) is a slow process. Median times to loss of sexual functions, as indicated in Table 3, vary between 5.8 and 12.9 months for morning erections and between 8.9 and 13.7 months for sexual activity between the CPA and FLU arms, respectively. The outcome with relation to almost all functions is more favourable as far as the time to their loss is concerned within the FLU-treated group of patients. However, these differences do not reach statistical significance. In this respect the study is inconclusive. The study, however, does not confirm previous studies with reported persistence of potency under treatment with FLU (Sogani et al, 1984; Lund and Rasmussen, 1988; Boccon-Gibod, 1998). The observation that some men remain potent under endocrine treatment is not new. Ellis and Grayhack (1963) reported that after castration, treatment with oestrogens or the combination of both 16 of 38 previously potent men remained potent over a prolonged period of time. The mechanism of maintained potency and sexual activity after castration as well as the mechanism of loss of potency clearly is not completely understood.

The investigators considered the fact that sexual partners are not involved in this protocol as one of the weaknesses of this study. For this reason, from time to time, partners who attended consultations were interviewed together with the patients. The sexual abilities and activity of the couple were confirmed by the partner on many occasions at the senior author’s institution.

In spite of this rather disappointing result, the use of what might be called ‘minimally invasive endocrine treatment’ with preservation of potency and a step-up scheme to more aggressive treatment once progression occurs, seems a realistic option based on the observation that half of the previously sexually active patients remain active for a year under FLU treatment and almost 9 months under treatment with CPA. This applies only if equal effectiveness with castration or an LHRH agonist could be proven in a prospective randomized study. Goldenberg et al (1995) have proposed intermittent endocrine treatment, a regimen that allows treatment-free periods of 4–6 months after similar periods of endocrine treatment. Intermittent endocrine treatment is at present subject to several large phase III studies. Because of the reversibility of the effect of anti-androgens and, especially utilizing the observation that the onset of impotence is a slow process under this type of treatment, pure anti-androgens and also steroidal anti-androgens may be ideal agents for use in intermittent treatment regimens of endocrine therapy. They might provide a 50% or more chance that men remain potent even during the active treatment. The occurrence of gynaecomastia in more than 40% of cases with the use of a pure anti-androgen is a problem that needs to be considered when taking these decisions (Schröder et al, 1997). Painful gynaecomastia was seen in 59–130 patients treated by FLU (45.4%) and in 10/134 patients treated with CPA (7.5%) $P = < 0.001$. Two patients in the FLU arm elected to discontinue treatment because of gynaecomastia. A correlation between gynaecomastia and loss of sexual functions was not seen. Another antiandrogen, which is available for clinical use in most countries, Bicalutamide®, may have a more favourable side-effect profile. However, the prevalence of gynaecomastia seems to be similar to FLU (Blackledge, 1996).

The problem of maintaining potency under endocrine treatment will become more relevant in the future, because prostate cancer is increasingly diagnosed at an earlier age. The diagnosis is more frequently based on an elevation of serum prostate-specific antigen (PSA) which has been shown to produce a lead time (the time diagnosis is moved forward with relation to the time of clinical diagnosis) of 6–10 years (Stenman et al, 1994). While the average age in this patient population amounts to 71 years the average age of men in populations diagnosed by the use of PSA driven diagnostics is clearly below 60. In addition, at this time adjuvant endocrine treatment and the issue of early versus delayed endocrine treatment of prostate cancer are still not fully understood (The Medical Research Council Prostate Cancer Working Party Investigators Group, 1997). Whatever developments in the near future will show: the age of men who are diagnosed to have prostate cancer will decrease and the relevance of the problem of sexual functioning will increase. With the recognition that maximal anti-androgen blockade has only minimal or no added value at all, and the expected prolonged periods of endocrine treatment with earlier diagnosis, it is necessary to investigate alternative treatment schemes. In spite of the observation that potency-related sexual functions decrease with time under anti-androgens, the observations presented in this paper give an opening for the development of new endocrine treatment strategies which take into consideration the quality of life related to sexual functioning.

### *Relevant background information*

Head on randomized comparison has not shown superiority of diethylstilboestrol (Pavone-Macaluso et al, 1986) and ethinyloestradiol (Jacobi et al, 1980) to CPA. Conclusive information on FLU monotherapy in comparison with standard treatment is not available, smaller trials show no difference with respect to time to progression and survival (Lund and Rasmussen, 1988; Boccon-Gibod, 1998).

### CONCLUSIONS

In this study of 294 men with metastatic prostate cancer and favourable prognostic factors sexual function was studied over time. A simple, five-item questionnaire was used. In comparing the pure anti-androgen FLU to the steroidal anti-androgen CPA

 © 2000 Cancer Research Campaign

(standard treatment arm) the original expectation that potency might be entirely preserved in the FLU arm was not confirmed. However, the paper allows a number of important conclusions. The initial rate of sexual dysfunction was rather high, probably higher than in the age-matched general population. The diagnosis of prostate cancer seems to have considerable impact on libido and sexual functioning in itself. This concept is supported by the fact that some men under both treatment regimens recovered sexual functions under treatment which were not reported at entry. In general, sexual function under FLU treatment was preserved in a higher proportion of men and for longer periods of time than under CPA. These differences, however, did not reach statistical significance. The observation that loss of potency under these treatment regimens is slow, may still give an opening for the improvement of sexuality-related quality of life in men with prostate cancer under endocrine treatment if the observations of this study are applied to new, ingenious treatment regimens.

### ACKNOWLEDGEMENTS

This publication was supported by grants numbers 5U10 CA11488-20 through 2U10 CA11488-28 from the National Cancer Institute (Bethesda, Maryland, USA). Its contents are solely the responsibility of the authors and do not necessarily represent the official views of the National Cancer Institute. Educational grants to the EORTC Genitourinary Group were provided by Schering AG, Berlin, Germany, and by Schering Plough Inc., Bloomfield, New Jersey, USA.

### REFERENCES

Agresti A (1990) *Categorical Data Analysis*. John Wiley & Sons: New York

Ahrens R (1990) Erfahrungen mit Cyproteronacetat bei Patienten mit Sexualdelinquenz. In: *Zur Therapie von sexuell Devianten*, Wille R, Schumacher W and Andrzejak N (eds), pp. 58–81. Diesbach Verlag: Berlin

Armitage P (1995) Test for linear trend in proportions and frequencies. *Biometrics* **11**: 375–386

Blackledge GP (1996) High-dose bicalutamide monotherapy for the treatment of prostate cancer. *Urology* **47**: 44–47

Boccon-Gibod L (1998) Are non-steroidal antiandrogens appropriate as monotherapy in advanced prostate cancer? *Eur Urol* **33**: 159–162

Dalesio O, Schröder FH, Peto R, and the Prostate Cancer Trialists' Collaborative Group (1995) Maximal androgen blockade in advanced prostate cancer: an overview of 22 randomised trials with 3289 deaths in 5710 patients. *Lancet* **346**: 265–269

Diokno AC, Brown MB and Herzog AR (1990) Sexual function in the elderly. *Arch Intern Med* **150**: 197

Eisenberger MA, Blumenstein BA, Crawford ED, Miller G, McLeod DG, Loehrer PJ, Wilding G, Sears K, Culkin DJ, Thompson IM, Bueschen AJ and Lowe BA (1998) Bilateral orchiectomy with or without Flutamide for metastatic prostate cancer. *N Engl J Med* **339**: 1036–1042

Ellis WJ and Grayhack JT (1963) Sexual function in aging males after orchiectomy and estrogen therapy. *J Urol* **89**: 895–899

Feldman HA, Goldstein I, Hatzichristou DG, Krane RJ McKinlay JB (1994) Impotence and its medical and psychosocial correlates: results of the Massachusettes male aging study. *J Urol* **151**: 54–61

Fitzpatrick JM, Kirby RS, Krane RJ, Adolfsson J, Newling DWW and Goldstein I (1998) Sexual dysfunction associated with the management of prostate cancer. *Eur Urol* **33**: 513–522

Frenken J (1978) SBS seksualiteit belevingsshalen; vragenlijst voor de man. In: Frenken J and Vennix P (eds), pp. 20. Swets & Zeitlinger: Lisse

Goldenberg SL, Bruchovsky N, Gleave ME, Sullivan LD and Akakura K (1995) Intermittent androgen suppression in the treatment of prostate cancer: a preliminary report. *Urology* **45**: 839–845

Helgason AR, Arver S, Adolfsson J, Dickman P, Granath F and Steineck G (1998) 'Potency'. The validation of information from a self-administrated questionnaire using objective measurements of night-time erections and test–retest reliability. *Br J Urol* **81**: 135–141

Jacobi GH, Altwein JE, Kurth KH, Basting R and Hohenfellner R (1980) Treatment of advanced prostatic cancer with parenteral Cyproterone acetate: a phase III randomised trial. *Br J Urol* **52**: 208–215

Kaplan EL and Meier P (1958) Non parametric estimation from incomplete observations. *J Am Statist Ass* **583**: 457–481

Lund F and Rasmussen F (1988) Flutamide versus Stilbestrol in the management of advanced prostatic cancer. *Br J Urol* **61**: 140–142

Mantel N (1966) Evaluation of survival data and two new rank order statistics arising in its consideration. *Cancer Chemother Rep* **50**: 163–170

Morley JE (1988) Impotence in older men. *Hospital Practice* **23**: 139

Pavone-Macaluso M, Voogt HJ de, Viggiano G, Barasolo E, Lardennois B, de Pauw M and Sylvester R (1986) Comparison of diethylstilbestrol, cyproterone acetate and medroxyprogesterone acetate in the treatment of advanced prostatic cancer: final analysis of a randomized phase III trial of the European Organization for Research on Treatment of Cancer Urological Group. *J Urol* **136**: 624–631

Robinson MRG, Smith PH, Richards B, Newling DWW, Pauw M de and Sylvester R (1995) The final analysis of the EORTC Genito-Urinary Group Phase III clinical trial (Protocol 30805) comparing orchidectomy, orchidectomy plus cyproterone acetate and low dose stilboestrol in the management of metastatic carcinoma of the prostate. *Eur Urol* **28**: 273–283

Schröder FH, Whelan P, Kurth KH, Sylvester R, de Pauw M and members of the EORTC Genitourinary Group (1997) Antiandrogens as monotherapy for metastatic prostate cancer: a preliminary report on EORTC protocol 30892. In: *Recent Advances in Prostate Cancer and BPH*, Proceedings of the IV Congress on Progress and Controversies in Oncological Urology (PACIOU IV), held in Rotterdam, The Netherlands, 11–13 April 1996. Schröder FH (ed), pp. 141–146, Parthenon: New York

Sogani PC, Minoo R, Vagaiwala R and Whitmore WS (1984) Experience with flutamide in patients with advanced prostatic cancer without prior endocrine therapy. *Cancer* **54**: 744–750

Stenman UH, Hakama M, Knekt P, Aromaa A, Teppo L and Leinonen J (1994) Serum concentrations of prostate specific antigen and its complex with 1-antichymotrypsin before diagnosis of prostate cancer. *Lancet* **344**: 1594–1598

The Medical Research Council Prostate Cancer Working Party Investigators Group (1997) Immediate versus deferred treatment for advanced prostatic cancer: initial results of the Medical Research Council Trial. *Br J Urol* **79**: 235–246

Voogt HJ de, Studer U, Schröder FH, Klijn JG, Pauw M de, Sylvester R and members of the EORTC Genito-Urinary Tract Cancer Cooperative Group (1998) Maximum androgen blockade using LHRH agonist Buserelin in combination with short-term (two weeks) or long-term (continuous) Cyproterone-Acetate (CPA) is not superior to standard androgen deprivation in the treatment of advanced prostate cancer. (Final analysis of EORTC GU group trial 30843.) *Eur Urol* **33**: 152–158

### APPENDIX

| Patients (%) | Main investigator |
|---|---|
| 11.3 | Mr P Whelan, St James Hospital, Leeds, United Kingdom |
| 10.0 | Dr ThM de Reijke, Academisch Medisch Centrum, Amsterdam, The Netherlands |
| 7.4 | Prof. Schröder |
| 4.8 | Prof. M Pavone-Macaluso, Universita di Palermo, Palermo, Italy |
| 4.5 | Dr J Mattelaer, St. Maarten Hospital, Kortrijk, Belgium |
| 4.2 | Dr RFP Van Velthoven, Institut Jules Bordet, Brussels, Belgium |
| 3.9 | Prof. D Newling, Academisch Ziekenhuis Der Vrije Universiteit, Amsterdam, The Netherlands |
| 3.9 | Prof. UE Studer, Inselspital, Bern, Switzerland |
| 3.5 | Dr M Brausi, Istituto Scientifico H.S. Raffaele, Milano, Italy |
| 3.2 | Prof. A Akdas, Marmara University Hospital, Istanbul, Turkey |
| 3.2 | Prof. L Denis, Algemeen Ziekenhuis Middelheim, Antwerpen, Belgium |

© 2000 Cancer Research Campaign

| | |
|---|---|
| 2.9 | Dr GON Oosterhof, St. Radboud University Hospital, Nijmegen, The Netherlands |
| 2.6 | Dr PPM Karthaus, Onze Lieve Vrouw Gasthuis, Amsterdam, The Netherlands |
| 2.6 | Dr MH Robinson, Weston Park Hospital, Sheffield, United Kingdom |
| 2.6 | Dr Janssen, Cliniques Universitaires Saint-Luc, Brussels, Belgium (*No Janssen there anymore) |
| 2.3 | Dr Bollack and Dr Saussine, Hospices Civils de Strasbourg, France (*Now it is Dr Jacqmin) |
| 2.3 | Mr J Hetherington, Princess Royal Hospital, Hull, United Kingdom |
| 1.9 | Dr RO Fourcade, Centre Hospitalier d'Auxerre, France |
| 1.6 | Prof. CL Cutajar, St Luke's Hospital, Malta |
| 1.6 | Dr JW Hoekstra, Groot Ziekengasthuis's Hertogenbosch, The Netherlands |
| 1.6 | Dr P Kil, St Elisabethziekenhuis, Tilburg, The Netherlands |
| 1.6 | Dr Fummer, Karl-Franzens-Universitaet Graz, Austria (*Now nobody from that Institute in the Gu) |
| 1.3 | Dr CGG Boeken-Kruger, Zuiderziekenhuis, Rotterdam, The Netherlands |
| 1.3 | Dr F Calais da Silva, Hospital Desterro, Amadora, Portugal |
| 1.3 | Dr JL Carneiro de Moura, Hospital Sta. Maria, Lisboa, Portugal |
| 1.3 | Dr R Fiala, Nemocnice Kromeriz, Kromeriz, Czech Republic |
| 1.3 | Dr O Koriakine, Medical Radiological Research Center, Obninsk, Russia |
| 1.3 | Dr W Oosterlinck, Universitair Ziekenhuis Gent, Gent, Belgium |
| 0.9 | Dr R Bastus-Piulats, Hospital de Mutua de Terrassa, Barcelona (Terrassa), Spain |
| 0.9 | Dr Gouveia, Hospital Dos Capuchos, Lisboa, Portugal |
| 0.9 | Prof. F Keuppens, Akademisch Ziekenhuis Vub, Brussels, Belgium |
| 0.9 | Dr A Nagy, University Medical School, Debrecen, Hungary |
| 0.9 | Dr H Waehre, Norwegian Radium Hospital, Oslo, Norway |
| 0.9 | Dr Bittard, CHR de Besançon, France (* Now there is nobody from that Institution in the GU) |
| 0.6 | Dr L Hoekx, Universitair Ziekenhuis Antwerpen, Edegem, Belgium |
| 0.6 | Prof. JP Sarramon, Chu de Purpan, Toulouse, France |
| 0.3 | Dr S Isorna, Hospital Nuestra Senora Del Pino, Las Palmas de Gran Canaria, Spain |
| 0.3 | Prof. Z Kirkali, Dokuz Eylul University School of Medicine, Izmir, Turkey |
| 0.3 | Dr KEJ Lantsoght, Lierse Ziekenhuizen, Lier, Belgium |
| 0.3 | Dr C Sternberg, San Raffaele Scientific Institute, Roma, Italy |
| 0.30 | Dr G Studler, Donauspital der Stadt Wien, Vienna, Austria |

© 2000 Cancer Research Campaign
